# Interactive Effects of Rising Temperature and Nutrient Enrichment on Aquatic Plant Growth, Stoichiometry, and Palatability

**DOI:** 10.3389/fpls.2020.00058

**Published:** 2020-02-12

**Authors:** Peiyu Zhang, Ayumi Kuramae, Casper H. A. van Leeuwen, Mandy Velthuis, Ellen van Donk, Jun Xu, Elisabeth S. Bakker

**Affiliations:** ^1^Department of Aquatic Ecology, Netherlands Institute of Ecology (NIOO-KNAW), Wageningen, Netherlands; ^2^Institute of Hydrobiology, Chinese Academy of Sciences (IHB-CAS), Wuhan, China; ^3^Department of Ecosystem Research, Leibniz-Institute of Freshwater Ecology and Inland Fisheries (IGB), Berlin, Germany; ^4^Department of Biology, Utrecht University, Utrecht, Netherlands

**Keywords:** herbivore, *Lymnaea stagnalis*, macrophyte, nitrogen, plant quality, phosphorus, *Vallisneria spiralis*, warming

## Abstract

The abundance and stoichiometry of aquatic plants are crucial for nutrient cycling and energy transfer in aquatic ecosystems. However, the interactive effects of multiple global environmental changes, including temperature rise and eutrophication, on aquatic plant stoichiometry and palatability remain largely unknown. Here, we hypothesized that (1) plant growth rates increase faster with rising temperature in nutrient-rich than nutrient-poor sediments; (2) plant carbon (C): nutrient ratios [nitrogen (N) and phosphorus (P)] respond differently to rising temperatures at contrasting nutrient conditions of the sediment; (3) external nutrient loading to the water column limits the growth of plants and decreases plant C:nutrient ratios; and that (4) changes in plant stoichiometry affect plant palatability. We used the common rooted submerged plant *Vallisneria spiralis* as a model species to test the effects of temperature and nutrient availability in both the sediment and the water column on plant growth and stoichiometry in a full-factorial experiment. The results confirmed that plants grew faster in nutrient-rich than nutrient-poor sediments with rising temperature, whereas external nutrient loading decreased the growth of plants due to competition by algae. The plant C: N and C: P ratios responded differently at different nutrient conditions to rising temperature. Rising temperature increased the metabolic rates of organisms, increased the nutrient availability in the sediment and enhanced plant growth. Plant growth was limited by a shortage of N in the nutrient-poor sediment and in the treatment with external nutrient loading to the water column, as a consequence, the limited plant growth caused an accumulation of P in the plants. Therefore, the effects of temperature on aquatic plant C:nutrient ratios did not only depend on the availability of the specific nutrients in the environment, but also on plant growth, which could result in either increased, unaltered or decreased plant C:nutrient ratios in response to temperature rise. Plant feeding trial assays with the generalist consumer *Lymnaea stagnalis* (Gastropoda) did not show effects of temperature or nutrient treatments on plant consumption rates. Overall, our results implicate that warming and eutrophication might interactively affect plant abundance and plant stoichiometry, and therefore influence nutrient cycling in aquatic ecosystems.

## Introduction

Climate change and eutrophication are altering the ecosystem functioning and services of shallow water bodies globally (IPCC, 2014; Steffen et al., 2015). In these shallow water bodies, aquatic plants are important components, as they can stabilize a clear water state (Hilt and Gross, 2008) and sustain high biodiversity (Declerck et al., 2005; Cronin et al., 2006). Due to ongoing eutrophication, the abundance of submerged aquatic plants has declined in many shallow water bodies (Sand-Jensen et al., 2000; Zhang et al., 2017), resulting in a shift from a stable clear water state with abundant submerged vegetation to a turbid stable state dominated by phytoplankton (Scheffer et al., 1993; Phillips et al., 2016). Global warming might also contribute to this collapse of submerged aquatic plants by promoting phytoplankton dominance (Mooij et al., 2007; Kosten et al., 2009). However, even without a collapse, more subtle changes may occur in aquatic plants if they are subjected to warming and eutrophication, which may still have far-reaching consequences for their role in the food web and for the cycling of nutrients in plant-dominated shallow water bodies. Particularly, alterations in plant stoichiometry, most commonly expressed as the carbon (C):nutrient [nitrogen (N) and phosphorus (P)] ratios, can affect plant decomposition and consumption by higher trophic levels (Sterner and Elser, 2002; Bakker et al., 2016).

Both warming (temperature rise) and eutrophication (nutrient enrichment) affect aquatic plant nutrient content and subsequent stoichiometry. Nutrient enrichment in the environment significantly increases the plant nutrient content (Dorenbosch and Bakker, 2011; Dülger et al., 2017), and decreases the C:nutrient ratios (Gu et al., 2016; Velthuis et al., 2017; Gu et al., 2018). However, studies on the impact of warming on aquatic plant C:nutrient ratios are scarce and yield contradictory results (Cross et al., 2015; Velthuis et al., 2017). The C:nutrient ratios might decrease (Ventura et al., 2008; Velthuis et al., 2017), remain unaltered (Zhang et al., 2016), or even increase (Kaldy, 2014; Zhang et al., 2016; Velthuis et al., 2018) in response to temperature rise. Similarly, field studies over a large temperature range also showed contradictory results, where the plant C:nutrient ratio either increased as temperature increased (Wang et al., 2015) (in the Tibetan Plateau, with minor anthropogenic disturbance), or decreased as temperature increased (Xia et al., 2014) (in eastern China, with high external nutrient loading to the water bodies). These contradictory impacts of temperature on the plant C:nutrient ratios might be caused by variation in nutrient conditions among experimental studies or field sites, suggesting that the impact of temperature rise on aquatic plant stoichiometry may depend on the nutrient availability in the environment. Natural systems are commonly subjected to both climate change and eutrophication (Jeppesen et al., 2010; Cross et al., 2015). Hence, there is an urgent need to study the combined effects of temperature rise and nutrient enrichment on aquatic plant stoichiometry.

In this study, we tested the interactive effects of rising temperature and nutrient enrichment of both the sediment and the water column on the growth and C:nutrient ratio of the common rooted submerged vascular aquatic plant *Vallisneria spiralis*, and assess the consequences for its palatability to a generalist herbivore. We cultured the plants at three different water temperatures (20, 24, and 28°C) and four distinct nutrient conditions (nutrient-poor and nutrient-rich sediments, with and without external nutrient loading) in a full-factorial design. Nutrient conditions were experimentally manipulated in the water column, the sediment, or both, because nutrient enrichment in eutrophic water bodies can result from external loading into the water column (Coppens et al., 2016), internal loading from the sediment (Fisher et al., 2005; Immers et al., 2015), or a combination thereof. We also monitored nutrient availability for the plants, and the development of competing primary producers (e.g. sestonic and periphytic algae) during the experiment.

**Figure 1:**
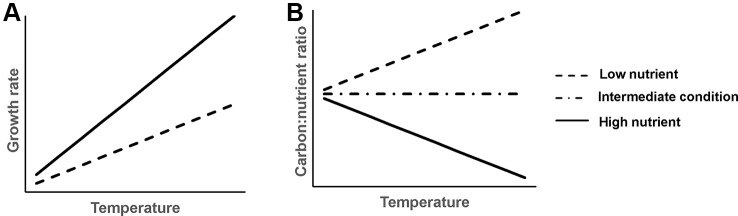
Schematic graph of hypothesized temperature effects on aquatic plant growth rate **(A)** and plant C:nutrient ratio **(B)** at different sediment nutrient conditions.

We formulated the following four hypotheses:

1. Plant growth rate increases faster with rising temperature in nutrient-rich than nutrient-poor sediments (**Figure 1A**). Generally, increasing temperature and nutrient availability both increase plant growth (Cross et al., 2015), hence, we would expect a synergistic effect of rising temperature and increasing nutrient availability in the sediment on plant growth. However, this only applies until plants reach their physiological temperature optimum or become light limited due to algal growth, after which plant growth is predicted to decline (Barko et al., 1982; Bakker et al., 2013).
2. Plant C:nutrient ratios respond differently to rising temperature at different sediment nutrient levels (**Figure 1B**). Specifically, at nutrient-rich sediment, the plant C:nutrient ratio is expected to decrease with rising temperature, as higher temperature can increase the mineralization rate of organic matter (Gudasz et al., 2010; Sobek et al., 2017), thereby leading to higher N and P availability for plants (Fisher et al., 2005; Alsterberg et al., 2012) and thus a lower plant C:nutrient ratio. At nutrient-poor sediment, however, plant C:nutrient ratios are expected to increase with rising temperature, as stimulated growth of plants can result in nutrient depletion. This could result in lower nutrient accumulation. Additionally, the plant physiology hypothesis predicts that plants may invest less N and P compared to C for their growth at higher temperature (Reich and Oleksyn, 2004; Toseland et al., 2013), resulting in higher plant C:nutrient ratios.
3. External nutrient loading to the water column can inhibit plant growth and decrease plant C:nutrient ratios. External nutrient loading could stimulate algae growth and inhibit growth of submerged plants (Barko et al., 1982; Bakker et al., 2013). Meanwhile, submerged plants accumulate nutrients and decrease C:nutrient ratios as the plant can take up nutrients from the water column (Carignan and Kalff, 1980; Rattray et al., 1991).
4. These hypothesized changes in plant stoichiometry due to temperature and nutrient enrichment are subsequently expected to affect plant palatability. A higher N content or lower C:N ratio in plant tissue generally corresponds to a higher plant consumption by herbivores (Cebrian and Lartigue, 2004; Bakker et al., 2016).

## Materials and Methods

### Plant Culturing

Our model species was *V. spiralis*, a rooted submerged aquatic plant that is widespread (Gupta, 2017) and relatively palatable for generalist consumers such as the pond snail *Lymnaea stagnalis* (Elger and Barrat-Segretain, 2004; Grutters et al., 2017). Rooted submerged aquatic plants can take up nutrients from both the sediment and the water column (Carignan and Kalff, 1980; Rattray et al., 1991; Christiansen et al., 2016), which allows detailed manipulation of nutrient conditions for our model species. Ten original plants of *V. spiralis* were obtained from a local garden center (Tuincentrum De Oude Tol, Wageningen, Netherlands) and planted in one aquarium to produce vegetative tillers. Seventy-two tillers (shoot length: 8.6 ± 2.0 cm, mean ± SD) were selected for the experiment. Each of these tillers was individually planted in a pot (top diameter 12.5 cm, bottom diameter 11 cm, and height 11 cm). Pots were each filled with 7 cm of sediment that was covered by a layer of 2 cm pure sand, to limit a nutrient flux between the sediment and the water column. Each pot was placed in a transparent cylindrical vase (inner diameter of 18 cm and height of 50 cm) filled with tap water (**Figure S1**).

A balanced full-factorial design was applied. Three temperature treatments were crossed with two sediment nutrient treatments and two external nutrient loading treatments that were applied to the water column (in total 12 treatments with n = 6). The three selected temperatures were 20, 24, and 28°C (steps of 4°C increase). The optimum temperature for *Vallisneria* growth is around 28°C (Barko et al., 1982; Bartleson et al., 2014), hence the increase in temperature along the selected temperature range implies increasing plant growth. The two sediment types consisted of nutrient-rich sediment (S1) with 100% artificial pond soil (Pokon Naturado, Veenendaal, Netherlands), and nutrient-poor sediment (S0) with 25% pond soil mixed with 75% sand (by volume). The pond soil contained 20% organic matter, with respectively 8.0 ± 0.48 mg g^−1^ (dry weight) and 1.1 ± 0.084 mg g^−1^ (dry weight) total N and total P (mean ± SE, n = 5). The two external nutrient loading treatments consisted of external nutrient loading to the water column (W1), and no external nutrient loading to the water column (W0). The nutrient solution was made by dissolving NH_4_NO_3_ and KH_2_PO_4_ powder in demineralized water. Nutrients were added weekly, simulating a high-level nutrient loading of 0.5 mg L^−1^ N and 0.05 mg L^−1^ P per week. The dosing level and ratio followed those of experiments in Sagrario et al. (2005); Jeppesen et al. (2007) and Coppens et al. (2016). These nutrient treatments are in the suitable range of the growth of the plant, as only high ammonia concentrations (> 5 mg L^−1^) can have toxic effects on the growth of submerged plants (Cao et al., 2004; Yu et al., 2015). To prevent the plants from being outcompeted by phytoplankton early during the experiment, the nutrient loading started half way (after 4 weeks) during the experiment and was subsequently applied every week until the end of the experiment.

The vases were placed in six aquaria (180 × 50 × 50 cm, l × w × h) which served as water baths to regulate the water temperature in the vases. Every aquarium contained 12 vases, and every two aquaria had the same temperature treatment. Vases with different nutrient treatments were randomly divided over the aquaria (see **Figure S1B** for a scheme of the experimental design). The experiment lasted for two months from October 6^th^ to December 5^th^ of 2016. The plants were first acclimated in their vases during the first week at 20°C, and subsequently assigned to the experimental temperatures. The day:night cycle was 16:8 h, and light intensity on the water surface during the day was 62 ± 17 μmol m^−2^ s^−1^ (mean ± SD, n = 72), a moderate light intensity (Middelboe and Markager, 1997), that was similar among treatments (*F*_1,11_ = 0.334, *p* = 0.97). Demineralized water was added twice a week to the vases to compensate for evaporation. The water level was elevated from 25 cm to 30 cm in all vases halfway the experiment, as the plants grew rapidly at the high temperature treatment and almost reached the surface.

Water quality parameters were measured four times during the experiment, and included conductivity, pH, chlorophyll a, alkalinity, NO_3_^−^, NH_4_^+^, and PO_4_^3−^ (the data are depicted in **Figure S2**). At the end of the experiment, the seston concentration (mainly phytoplankton) was quantified by filtering a known volume of water (adapted to the concentration of the phytoplankton) over pre-weighed GF/F filters (Whatman, Maidstone, UK). Filters were thereafter dried in the oven at 60°C for 48 h and reweighed. The seston concentration was expressed as mg dry weight per liter of water (**Figure S3** and **Table S1**). Periphyton growth was quantified by fixing a transparent polypropylene strip (21 × 2 cm, l × w) in each vase at the start of the experiment, and collected again at the end of the experiment. The periphyton dry weight (μg dry weight per cm^2^ area) was determined by cutting a certain size of the strip (from 4 to 21 cm^2^, determined by the density of periphyton), cleaning it with a toothbrush in a beaker with demineralized water and filtering the water over pre-weighed filters (Whatman, Maidstone, UK). The filters were dried in the oven at 60°C for 48 h and weighed, the change in dry weight of the filter allowed quantification of the dry weight of the periphyton (**Figure S3** and **Table S1**). To determine the sediment nutrient availability for the plants, sediment porewater was sampled in each pot using rhizons (Rhizosphere, Wageningen, Netherlands) at the end of the experiment. The porewater was then analyzed for total dissolved inorganic nitrogen (DIN: including N from NH_4_^+^, NO_2_^−^ and NO_3_^−^) and P-PO_4_^3−^ concentrations on an auto analyzer (QuAAtro method, Seal Analytical, Fareham, UK) (**Figure S3** and **Table S1**).

At the end of the experiment, about 0.4 g fresh plant material from each pot was collected for the feeding trials with the aquatic snails. The rest of the plant material was harvested to quantify dry biomass and C:N:P stoichiometry. Shoots and roots were separated, cleaned carefully and oven-dried at 60°C for 48 h. Plant relative growth rate was calculated according to the equation: Relative growth rate = (ln W_f_ – ln W_i_)/days (Hunt, 1982); with W_i_ = initial dry weight and W_f_ = final dry weight, where W_f_ is the sum of shoot and root biomass (including the estimated weight of the plant parts used for feeding trials). Plant initial dry weight was determined by drying and weighing 10 spare plants before the start of the experiment.

Each dried plant sample was ground individually in a 2 ml tube on a ball mill Tissuelyser II (QIAGEN, Hilden, Germany). Plant C and N were determined on an elemental NC analyzer (FLASH 2000, Thermo Scientific, Waltham, MA, USA). P content was determined according to Murphy and Riley (1962) by incinerating and digesting the organic P, and then measuring the dissolved phosphate concentration on an Auto Analyzer (QuAAtro method, Seal Analytical, Fareham, UK).

### Snail Culturing and Palatability Test

We tested for variation in palatability among the cultured plants using a generalist consumer, the pond snail *L. stagnalis*. This species can feed on a large variety of aquatic plants, and is frequently used as a model species for testing aquatic plant palatability (Elger and Barrat-Segretain, 2002; Elger and Barrat-Segretain, 2004; Grutters et al., 2017; Zhang et al., 2018a). We hatched snails from egg clusters from a pond of NIOO-KNAW (51°59'16.8”N, 5°40'24.7”E, Wageningen, Netherlands). Juvenile snails were reared for 2 months in buckets at 20°C that were filled with tap water and constantly aerated, under a day:night cycle of 16:8 h. We fed snails commercially obtained lettuce five times per week. Fish food (Velda, Gold Sticks Basic Food, Netherlands) and chalk were supplied weekly as food and mineral supplements providing other nutrients. Snails of similar size (shell length 30.4 ± 0.9 mm, mean ± SD, n = 62) were selected for the palatability tests.

The palatability tests followed the protocol developed by Elger and Barrat-Segretain (2002; 2004). This test measures how much plant material is consumed by one individual snail over a certain time, using no-choice feeding trials. The snails were individually placed in a beaker (volume of 500 ml) with 375 ml tap water for 24 h without food before the feeding trials. From each vase, approximately 0.2 g wet weight of fresh plant leaves was harvested, cleaned to remove periphyton, and offered to each snail. This was the maximum amount of plant material that one snail could eat in 1 day as determined in pre-trials. As control, another 0.2 g leaves from the same vase was placed in a beaker without a snail to monitor possible weight changes in plant material due to decomposition or growth over 24 h. Each beaker was covered with a mesh to prevent the snail from escaping. After the feeding trials, leftover plant material was weighed and dried in the oven at 60°C for 48 h and weighed again. The snails were frozen, dried in the oven at 60°C with their shell separated from the soft body part, and weighed. Plant dry matter content was determined as the dry weight divided by the wet weight and expressed as percentage, using the control portion of the plant. Plant dry matter content can be used to indicate plant toughness, and has been shown to negatively correlate with aquatic plant palatability (Elger and Willby, 2003). Plant palatability, indicated by plant relative consumption rate (RCR) (mg g^−1^ d^−1^), was calculated according to Elger and Barrat-Segretain (2002): RCR = [(C_fd_/C_iw_) * F_iw_ − F_fd_]/S_d_/1 day, where C_fd_ is the final dry weight of the control plant, C_iw_ is the initial wet weight of the control plant, F_iw_ is the initial wet weight of the feeding trial plant, F_fd_ is the final dry weight of the feeding trial plant, and S_d_ is the snail dry weight without shell.

### Data Analysis

In five vases plants died during the experiment, which were excluded from the dataset (dead plants were spread over the treatments: one in the 20°C W1S1 treatment, one in 24°C W0S0, one in 24°C W1S0, one in 24°C W0S1, and one in the 28°C W1S0 treatment). This resulted in 67 individual plants being available for the analysis of four plant growth parameters (plant shoot biomass, root biomass, relative growth rate, and root:shoot ratio), three plant elemental compositions (plant C, N, and P content), and three plant stoichiometry traits (C:N, C:P, and N:P ratio). The palatability test was performed on 62 individual plants, as another five vases (mainly at low temperatures and low nutrient levels: four at 20°C W0S0 and one at 24°C W1S0) did not contain enough plant material for the feeding trials as plants grew slowly under these conditions and were therefore excluded. Linear mixed-effect models, using R package *nlme* (Pinheiro et al., 2017), were used to analyze the effects of temperature, nutrient treatment, and their interactions on all the parameters. Aquarium was set as a random factor in all the models to account for the dependency structure in our experimental blocked design. QQplot and residual plot were used to test the normality of data. If data were not normally distributed, data were transformed (data transformation is added in **Table 1**). Estimated marginal means and estimated marginal means of linear trends were calculated after each linear mixed-effects model test to compare the difference of the means and slopes among the four nutrient treatments, respectively, using R package *emmeans* (Lenth et al., 2019).

**Table 1:** Effects of temperature, nutrient treatment, and their interactions on plant growth, elemental composition, stoichiometry, and plant palatability. Effects were analyzed by linear-mixed effect models. Data transformation to meet model requirements is indicated.

Category	Parameters	Factors	df	*F*	*p*-value	Means comparison	Slopes comparison
**Plant growth**	**Shoot biomass**	Temp	1, 4	29.36	**0.0056**	a, a, b, b	A, A, B, AB
		Nutrient	3, 55	25.79	**<0.0001**		
		Temp × Nutrient	3, 55	8.80	**0.0001**		
	**Root biomass**	Temp	1, 4	16.63	**0.0151**	a, a, b, a	A, A, A, A
		Nutrient	3, 55	5.97	**0.0013**		
		Temp × Nutrient	3, 55	2.28	0.0890		
	**Relative growth rate**	Temp	1, 4	71.39	**0.0011**	a, a, b, b	A, A, B, AB
		Nutrient	3, 55	28.29	**<0.0001**		
		Temp × Nutrient	3, 55	6.71	**0.0006**		
	**log(Root : Shoot ratio + 0.001)**	Temp	1, 4	34.90	**0.0041**	b, b, a, a	A, A, A, A
		Nutrient	3, 55	31.93	**<0.0001**		
		Temp × Nutrient	3, 55	0.63	0.5963		
**Plant nutrient content**	**Carbon**	Temp	1, 4	31.10	**0.0051**	b, a, b, a	AB, A, B, AB
		Nutrient	3, 55	12.40	**<0.0001**		
		Temp × Nutrient	3, 55	3.20	**0.0291**		
	**log(Nitrogen)**	Temp	1, 4	1.44	0.2962	a, b, b, c	AB, A, B, AB
		Nutrient	3, 55	90.93	**<0.0001**		
		Temp × Nutrient	3, 55	3.07	**0.0351**		
	**log(Phosphorus)**	Temp	1, 4	15.23	**0.0175**	b, c, a, bc	B, B, AB, A
		Nutrient	3, 55	11.60	**<0.0001**		
		Temp × Nutrient	3, 55	6.70	**0.0006**		
**Plant stoichiometry**	**C:N ratio**	Temp	1, 4	2.33	0.2016	c, b, b, a	A, A, A, A
		Nutrient	3, 55	95.06	**<0.0001**		
		Temp × Nutrient	3, 55	2.89	**0.0434**		
	**C:P ratio**	Temp	1, 4	14.10	**0.0199**	b, a, c, ab	A, A, AB, B
		Nutrient	3, 55	12.72	**<0.0001**		
		Temp × Nutrient	3, 55	5.51	**0.0022**		
	**sqrt(N:P ratio)**	Temp	1, 4	1.87	0.2436	a, b, c, c	A, A, B, B
		Nutrient	3, 55	127.07	**<0.0001**		
		Temp × Nutrient	3, 55	14.55	**<0.0001**		
**Plant palatability**	**RCR**	Temp	1, 4	4.04	0.1149	a, a, a, a	A, A, A, A
		Nutrient	3, 55	1.43	0.2450		
		Temp × Nutrient	3, 55	0.56	0.6414		

**Figure 2:**
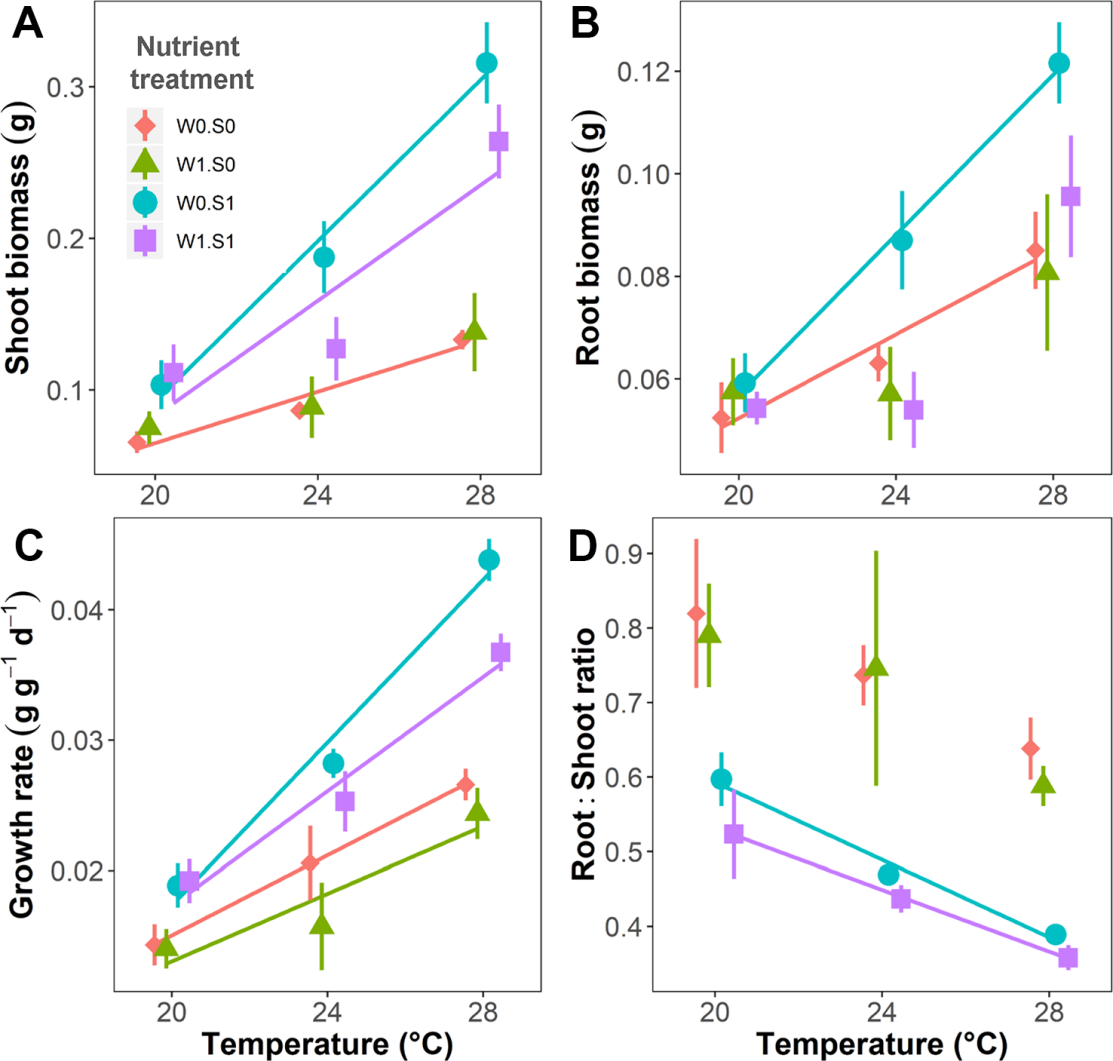
Temperature effects on plant growth parameters indicated per nutrient treatment. **(A)** Plant shoot biomass, **(B)** root biomass, **(C)** relative growth rate, and **(D)** root:shoot ratio. S1 indicates nutrient-rich sediment, S0 indicates nutrient-poor sediment, W1 indicates with external nutrient loading to the water, and W0 indicates without external nutrient loading. A solid line indicates *p* < 0.05, and no line is drawn when *p* > 0.05. Vertical bars are standard errors (n = 6).

**Figure 3:**
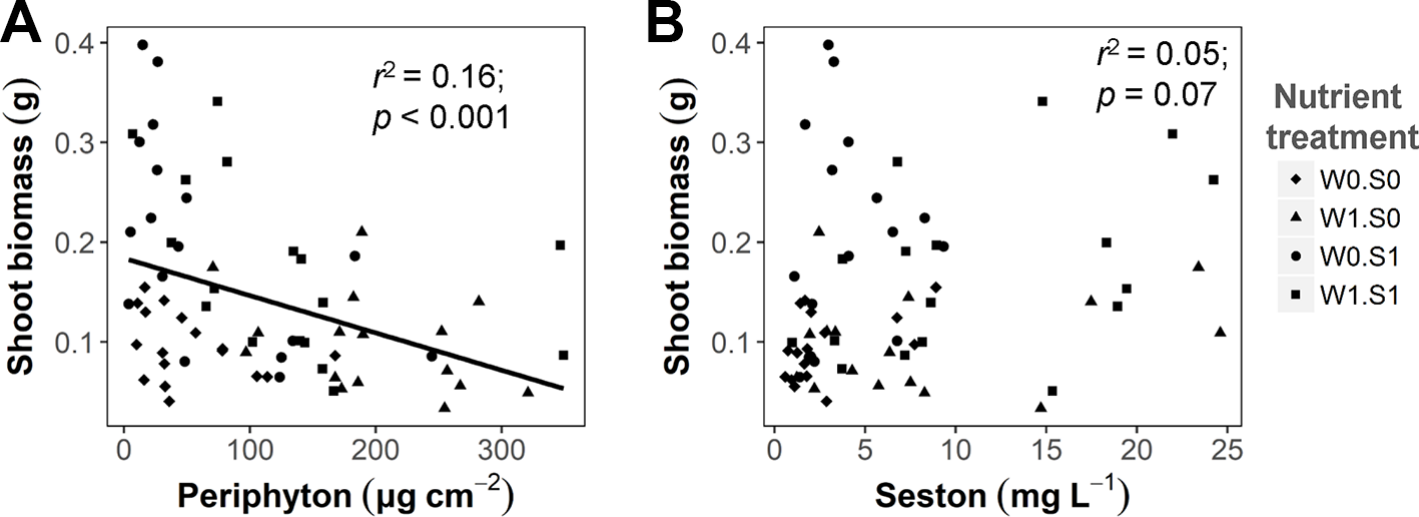
The relationship between algae growth and plant shoot biomass at the end of the experiment. **(A)** Periphyton biomass density (dry weight) and plant shoot biomass (dry weight per vase); **(B)** Seston concentration (dry weight) and plant shoot biomass. Linear regression test results are shown in the figures. See caption of **Figure 2** for an explanation of the abbreviations of the nutrient treatments.

**Figure 4:**
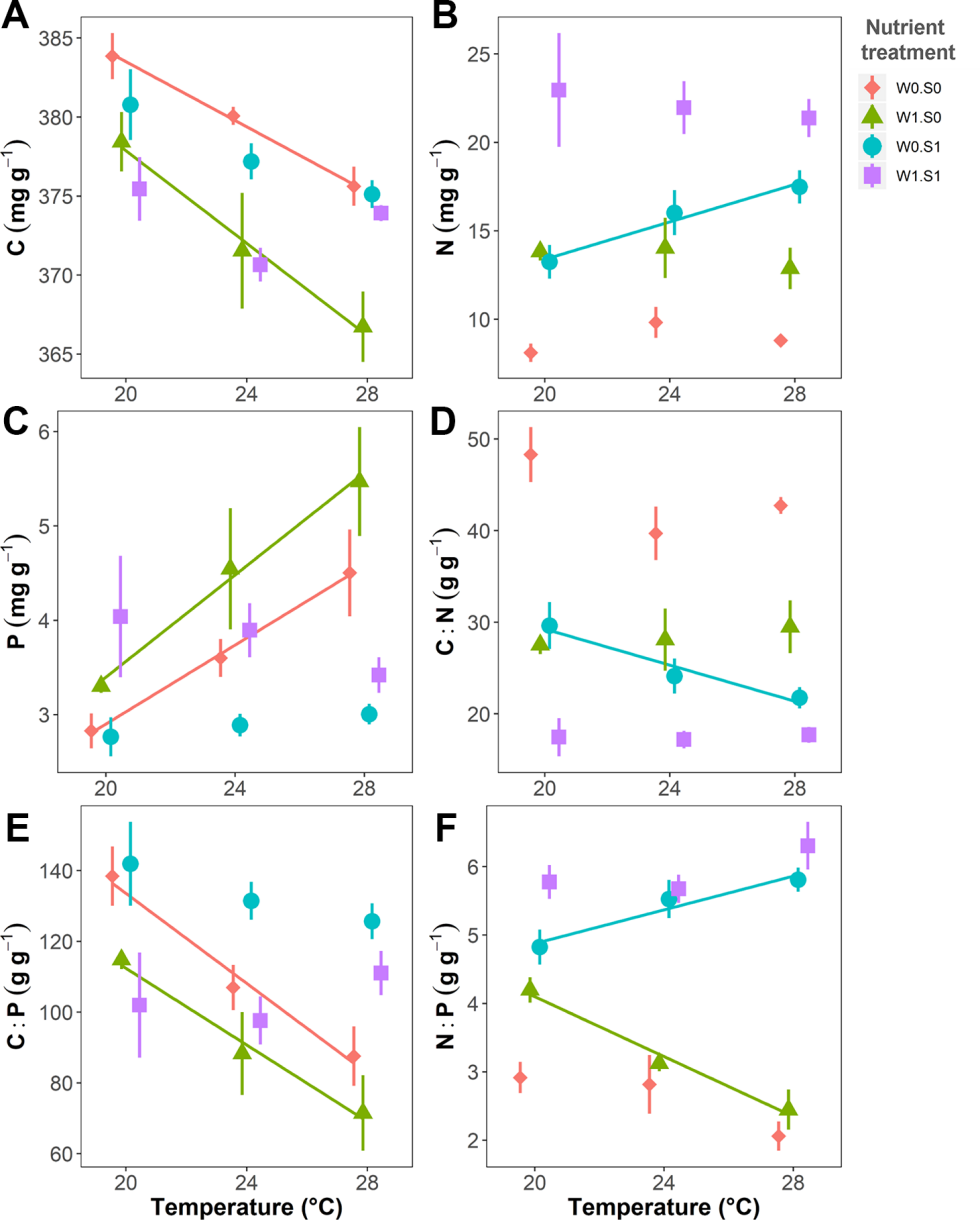
Temperature effects on plant elemental composition (C, N, and P contents) and stoichiometry (C:N, C:P, and N:P ratio) in dry weight indicated per nutrient treatment. **(A)** Plant C content, **(B)** N content, **(C)** P content, **(D)** C:N ratio, **(E)** C:P ratio, and **(F)** N:P ratio. Nutrient treatments are as indicated in **Figure 2**. A solid line indicates *p* < 0.05, and vertical bars are standard errors (n = 6).

**Figure 5:**
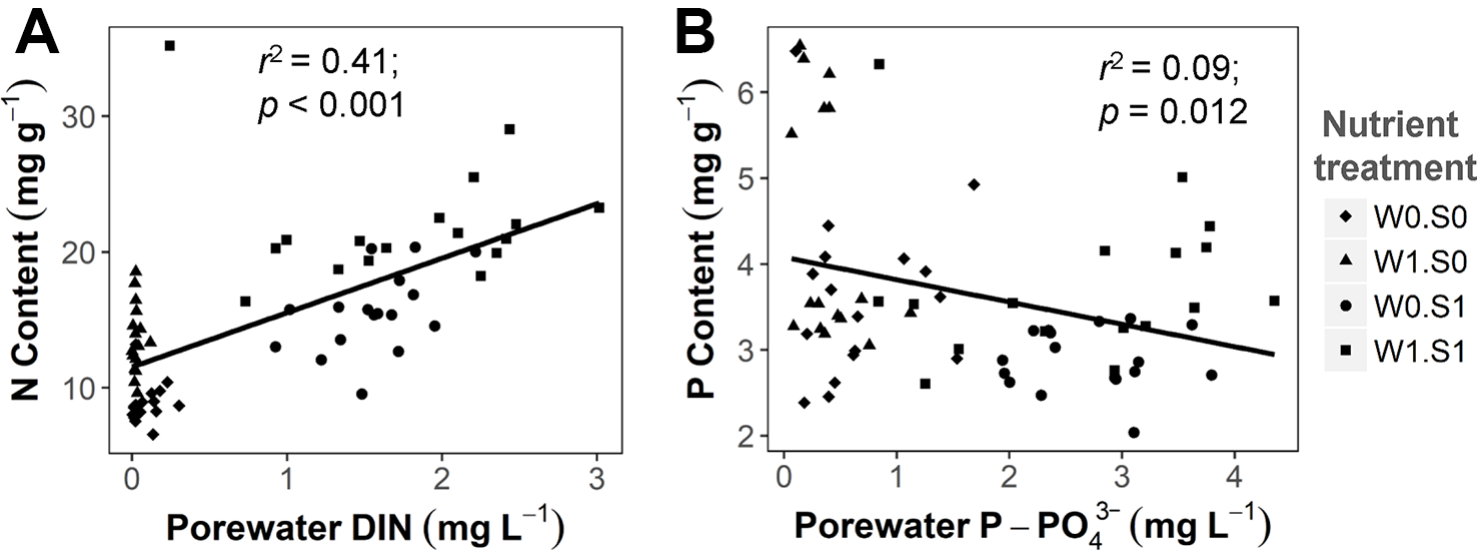
The relationship between sediment porewater nutrient concentrations and plant nutrient contents. **(A)** porewater DIN concentration and plant N content, DIN indicates total dissolved inorganic nitrogen (including N from NH_4_^+^, NO_2_^−^, and NO_3_^−^). **(B)** porewater P-PO_4_^3−^ concentration and plant P content. Linear regression test results are shown in the figures. See caption of **Figure 2** for an explanation of the abbreviations of the nutrient treatments.

**Figure 6:**
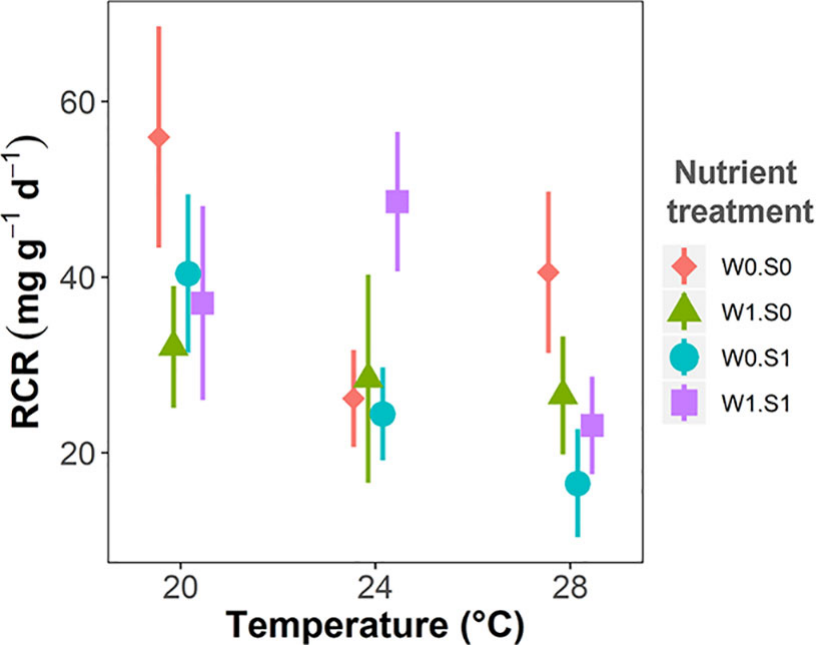
Temperature effects on plant palatability to the pond snail *L. stagnalis* expressed as relative consumption rate (RCR), indicated per nutrient treatment. Nutrient treatments are as indicated in **Figure 2**. Vertical bars are standard errors (n = 6).

Means and slopes comparison among the four nutrient treatments were performed after each linear mixed-effect model test. Different letters indicate differences among the four nutrient treatments in an order of W0.S0, W1.S0, W0.S1 and W1.S1, the same order as presented in **Figures 2**–**6**. “Temp” represents temperature treatment. “Nutrient” indicates the four nutrient treatments. “RCR” represents plant relative consumption rate. “log” and “sqrt” indicate the data are natural log and square root transformed respectively. Bold numbers indicate p < 0.05.

After the global test, temperature effects were also separately tested in linear mixed-effect models in all four nutrient treatments (W0.S0, W1.S0, W0.S1, and W1.S1), with temperature as a fixed factor and aquarium as a random factor. These tests provided the formulas, *r*^2^ values (conditional coefficient of determination) and *p*-values as presented directly in the figures. Simple linear regression tests (R function “*lm*”) were applied to test the correlation between sediment nutrient concentration and plant nutrient content, and between plant palatability and plant dry matter content, elemental composition, and stoichiometry.

A structural equation model (SEM) was constructed to summarize the effects of temperature, sediment, and external nutrient loading treatments on the growth and elemental composition of the plant. This allowed assessing the complete graphical network of the interactions and relationships, with the directions of paths in the SEM diagram indicating causal influences (Rosseel, 2012). Three indices of model fit were used with conventional significance thresholds to assess the overall fit of the SEM, with the χ^2^ *p* value (*p* > 0.05), the standardized root mean squared residual (SRMR ≤ 0.08), and the comparative fit index (CFI ≥ 0.95) (Hu and Bentler, 1999). Model selection was done by removing non-significant paths from the *a priori* model with all the possible interactions included (e.g. seston was removed). A maximum likelihood estimation (ML) with robust standard errors was applied to correct for the deviation of normality of the continuous variables (Rosseel, 2012). All SEM procedures were conducted with the *lavaan* (version 0.6-3) package in R (Rosseel, 2012). All analysis were performed in R version 3.5.3 (R Development Core Team, 2019). The code for building the SEM is provided in the **Supplementary Material S1**.

## Results

### Plant Growth and Culturing Conditions

Temperature and nutrient treatments all affected the growth parameters of the plants, and the effects of temperature depended on the nutrient treatments (**Figure 2** and **Table 1**). Rising temperature significantly increased plant shoot biomass, root biomass, and relative growth rate in the treatments without external nutrient loading (W0S0 and W0S1). Plant shoot biomass and growth rate both increased faster with rising temperature in nutrient-rich (W0S1) than in nutrient-poor (W0S0) sediment treatments (slopes comparison, **Figure 2** and **Table 1**). With external nutrient loading, plant growth rate still increased with rising temperature, whereas plant root biomass was not affected by temperature (W1S0 and W1S1). Plant shoot biomass only increased with rising temperature in the treatment with nutrient-rich sediment (W1S1). Plant shoot biomass, root biomass and relative growth rate were all significantly higher in nutrient-rich sediment than nutrient-poor sediment (**Figure 2** and **Table 1**). The plant root:shoot ratio, an indicator of plant biomass allocation, was also affected by the treatments. Rising temperature significantly decreased the plant root:shoot ratio in the nutrient-rich sediment treatments (W0S1 and W1S1), but not in the nutrient-poor sediment treatments (W0S0 and W1S0) (**Figure 2** and **Table 1**). Plant root:shoot ratio decreased in the nutrient-rich sediment, whereas external nutrient loading had no significant effects.

Competing primary producers were also influenced by the experimental treatments. Rising temperature significantly increased the seston concentration in the treatment with external nutrient loading and nutrient-rich sediment (W1S1), but not in the other nutrient treatment (**Figure S3** and **Table S1**). The seston concentration increased with external nutrient loading, but was not affected by the sediment nutrient treatment. Periphyton concentrations were only affected by external nutrient loading, not by nutrients in the sediment or water temperature (**Figure S3** and **Table S1**). There was a negative correlation between the periphyton concentration and plant shoot biomass (**Figure 3A**), but no significant correlation between the seston concentration and plant shoot biomass (**Figure 3B**). A rising temperature significantly increased the porewater DIN concentration in the W0S1 treatment, but not in the other nutrient treatments. There were no temperature effects on the porewater P-PO_4_^3−^ concentration (**Figure S3** and **Table S1**). Both sediment porewater DIN and P-PO_4_^3−^ concentrations were much higher in the nutrient-rich sediment than the nutrient-poor sediment (**Figure S3** and **Table S1**).

### Plant Elemental Composition and Stoichiometry

Temperature and nutrient treatments all affected the plant elemental composition and stoichiometry, and the effects of temperature depended on the nutrient treatments (**Figure 4** and **Table 1**). The relative variance of the plant C content (CV, coefficient of variation, 1.6%) was much lower than the variance of the plant N (CV, 38.0%) and P content (CV, 27.0%). Therefore, the temperature and nutrient enrichment effects on C:N and C:P ratios were mainly determined by the effects on N and P content, respectively. Rising temperature significantly decreased the plant C content in the treatments with nutrient-poor sediment (W0S0 and W1S0), not in nutrient-rich sediment (W0S1 and W1S1) (**Figure 4** and **Table 1**). Plant C content decreased with external nutrient loading, but was unaffected by the sediment nutrient treatment.

Rising temperature increased plant N content and therefore decreased plant C:N ratios in the treatment with nutrient-rich sediment but without external nutrient loading (W0S1). However, there were no responses in the other nutrient treatments (**Figure 4** and **Table 1**). Plant N content increased and plant C:N ratio decreased in the nutrient-rich sediment treatment, and with external nutrient loading.

Rising temperature significantly increased plant P content and decreased plant C:P ratio in the nutrient-poor sediment treatments (W0S0 and W1S0), but not in the nutrient–rich sediment treatments (W0S1 and W1S1) (**Figure 4** and **Table 1**). Plant P content increased and C:P ratio decreased with external nutrient loading, but plant P content decreased and C:P ratio increased in nutrient-rich sediment. The plant N content was positively correlated with the porewater DIN concentrations (**Figure 5A**). In contrast, the plant P content was negatively correlated with the porewater P-PO_4_^3−^ concentrations (**Figure 5B**).

Rising temperature significantly decreased the plant N:P ratio in the treatment with only external nutrient loading (W1S0), and increased the plant N:P ratio in the treatment with only enriched sediment (W0S1), whereas there were no effects in the other nutrient treatments (**Figure 4** and **Table 1**). The plant N:P ratio increased in nutrient-rich sediment, and with external nutrient loading.

### Plant Palatability

Plant palatability, measured as the relative consumption rate (RCR), ranged from 0 to 77 mg g^−1^ d^−1^, irrespective of temperature or nutrient treatments (**Figure 6** and **Table 1**). Plant palatability did not correlate with plant dry matter content, nor with any of the measured plant elemental compositions or stoichiometric parameters. (Linear regression testing palatability (RCR) with respectively dry matter content, *r^2^* = 0.04, *p* = 0.14; C content, *r^2^* = 0.01, *p* = 0.498; N content, *r^2^* = 0.01, *p* = 0.577; P content, *r^2^* = 0.02, *p* = 0.324; C:N ratio, *r^2^* = 0.01, *p* = 0.551; C:P ratio, *r^2^* = 0.002, *p* = 0.737; N:P ratio, *r^2^* = 0.01, *p* = 0.544.)

### Complete Diagram Interactions

**Figure 7:**
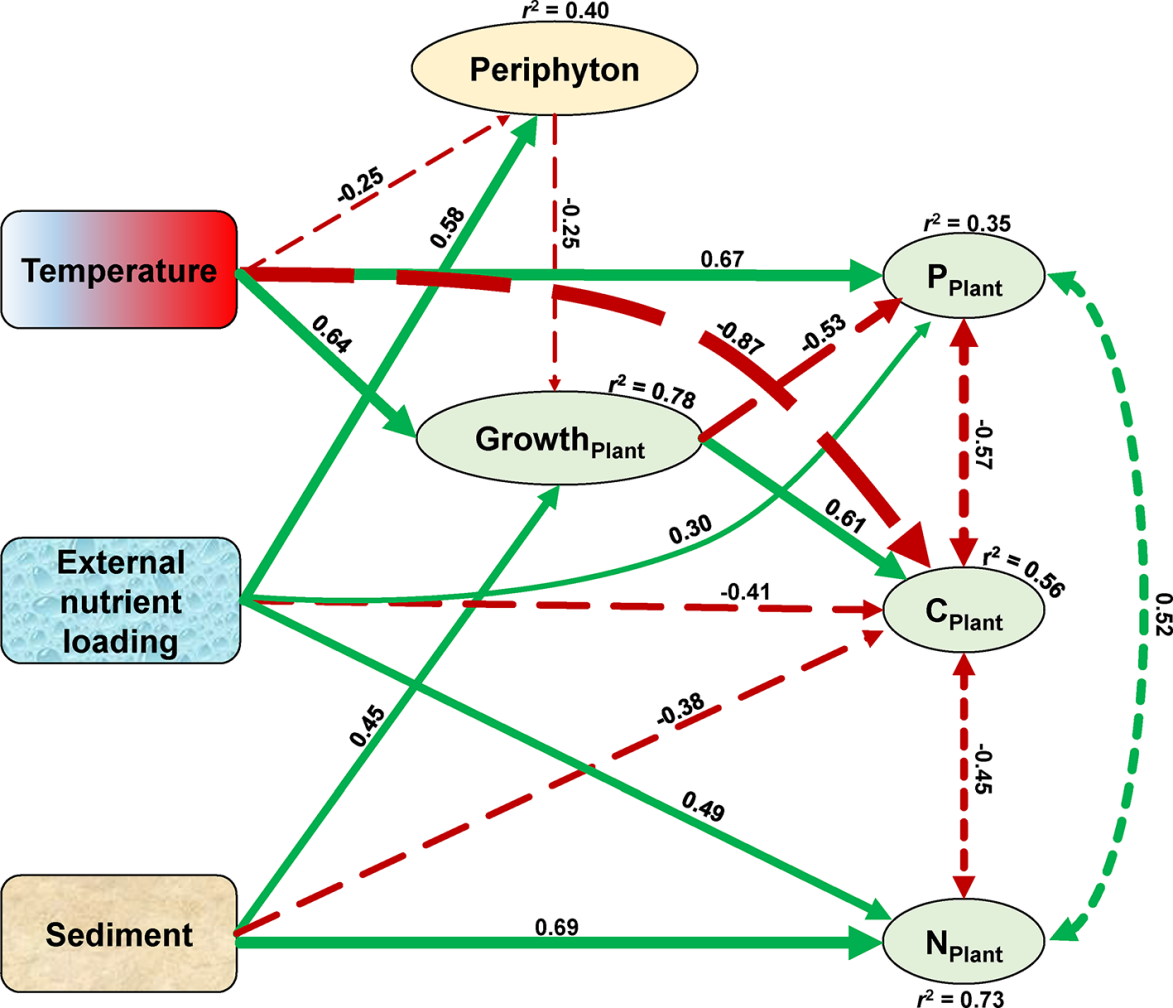
Structural equation model (SEM) of temperature, sediment, and external nutrient loading treatment effects on the growth and elemental compositions of the plant. Exogenous variables are indicated by rounded rectangles, and endogenous variables are represented by ovals. Coefficients of determination (r^2^) are shown for all endogenous variables. Numbers adjacent to arrows are standardized path coefficients and indicative of the effect of the relationship. Positive and negative effects among variables are depicted by green solid and red long-dashed arrows, respectively, with arrow thicknesses proportional to the strength of the relationship. Covariance between the plant elements are depicted by dashed double-headed arrows. The covariance between N and P content of the plant marginally significant at *p* = 0.06, all other relationships in the model are significant at *p* < 0.01. The model satisfied each of the three model fit criteria with significant χ^2^ of *p* = 0.35, standardized root mean squared residuals of 0.04, and comparative fit index values of 0.997.

The SEM confirmed that temperature, the sediment, and external nutrient loading treatments all affected the growth and stoichiometry of the plants both directly and indirectly (**Figure 7**). The growth of the plant (overall explanation *r*^2^ = 0.78) was enhanced by rising temperature (standardized path coefficient, SPC = 0.64) and nutrient-rich sediment (SPC = 0.45), whereas external nutrient loading indirectly inhibited the growth of the plant (SPC = −0.25) by increasing the growth of periphyton (SPC = 0.58). Plant C content (overall explanation *r*^2^ = 0.56) decreased with rising temperature (SPC = −0.87), external nutrient loading (SPC = −0.41), and in nutrient-rich sediment (SPC = −0.38), but increased with the growth of the plant (SPC = 0.61). Plant N content (overall explanation *r*^2^ = 0.73) increased with external nutrient loading (SPC = 0.49) and in nutrient-rich sediment (SPC = 0.69). Plant P content (overall explanation *r*^2^ = 0.35) was directly enhanced by rising temperature (SPC = 0.67) and external nutrient loading (SPC = 0.30), whereas the growth of the plant decreased the P content (SPC = −0.53). In addition, plant C content negatively covaried with plant N (SPC = −0.45) and P (SPC = −0.57) content, and plant N content positively covaried with plant P content (SPC = 0.52) (**Figure 7**).

## Discussion

We tested how temperature rise and nutrient enrichment interactively affected aquatic plant growth, stoichiometry, and palatability. Temperature effects on plant growth and stoichiometry were highly dependent on the nutrient conditions in the environment. Effects depended on whether nutrients were available in the sediment or in the water column. Plant growth rates increased faster with rising temperature in nutrient-rich than nutrient-poor sediments, which confirms our first hypothesis. However, plant C:N ratios decreased in nutrient-rich sediments and the plant C:P ratio decreased in nutrient-poor sediments, which is inconsistent with our second hypothesis. Temperature effects on plant stoichiometry were not only dependent on the specific nutrients in the environment, but also depended on plant growth. External nutrient loading in the water column inhibited plant growth due to enhanced growth of periphyton, and plant C:N and C:P ratios all decreased with external nutrient loading, which confirms the third hypothesis. Even though plant stoichiometry changed due to the temperature and nutrient treatments, we did not detect changes in plant palatability and thus reject the fourth hypothesis. We discuss the mechanisms and implications of our findings below in more detail.

### Plant Growth

Rising temperature can stimulate the growth of aquatic plants in their suitable temperature range, as shown in a large variety of aquatic plant species (Barko et al., 1982; Madsen and Brix, 1997; Kaldy, 2014; Velthuis et al., 2017). However, effects of rising temperature on plant growth also depend on the availability of nutrients to realize growth (Cross et al., 2015). In our study, plant relative growth rates increased faster at high sediment nutrient availability, demonstrating an interactive effect of temperature and nutrient enrichment on plant growth. However, this effect depended on where the added nutrients were available, in the sediment or in the water column, as external nutrient loading to the water column inhibited the growth of plants. Although *V. spiralis* can take up the added nutrients from the water column, algae can do this as well (Van Donk and Van De Bund, 2002; Yu et al., 2015), and more efficient than *V. spiralis*, indicated by the enhanced seston and periphyton biomass observed in our experiment with external nutrient loading. Algae can compete with the plants for nutrients as well as light. As a result, *V. spiralis* profited from nutrient enrichment in the sediment, but suffered from competition by algae, in particular periphyton, under external nutrient loading (**Figure 3**), as also illustrated in the SEM (**Figure 7**). The periphyton biomass densities observed in our study correspond to a reduction in light availability of approximately 20% at the high end of the observed periphyton densities (up until approximately 300 µg cm^−2^), if periphyton directly grows on the leaves of the plants (Hidding et al., 2016). Therefore, in our study, *V. spiralis* suffered from competition by periphyton, which may have to a limited extent resulted from shading, but it is not possible to pinpoint whether light competition or nutrient competition was driving the observed effect. The plant root:shoot ratio decreased with rising temperature and nutrient enrichment in the sediment. Possibly, plant nutrient uptake efficiency increases at higher temperatures, and thus plants invest less biomass in root formation (Barko and Smart, 1981; Barko et al., 1982; Riis et al., 2012). Furthermore, in nutrient-rich sediments, there were more nutrients available, hence plants allocated less biomass to roots (Olsen and Valiela, 2010). These shifts in plant root:shoot ratio can be explained by the optimal partitioning theory, which indicates that plants invest more biomass in tissue suitable to take up nutrients during growth if there is nutrient limitation (Bloom et al., 1985), and is an adaptive strategy for plants to cope with environmental changes.

### Plant Nutrient Uptake

In our experiment, the pH varied from 7 to 10 (**Figure S2**), indicating that the major C source for the plant was bicarbonate (HCO_3_^−^) (Maberly and Gontero, 2017), and *V. spiralis* could utilize bicarbonate as its main C source in this condition (Iversen et al., 2019). The alkalinity (mainly represented by bicarbonate, **Figure S2**) was almost always above 1.0 meq L^−1^ which indicates that the growth of *V. spiralis* might not be limited by C availability during the experiment (Vestergaard and Sand-Jensen, 2000a; Vestergaard and Sand-Jensen, 2000b). The relatively small variation of C content in the plants also suggests this.

Generally, plant N content is related to the environmental N availability (Cronin and Lodge, 2003; Demars and Edwards, 2007; Cao et al., 2011; Zhang et al., 2019). In our study, plant N content increased both in nutrient-rich sediment and with external nutrient loading. This indicates that the plant could take up N from both the sediment and the water column, which is consistent with previous studies (Cao et al., 2011; Gu et al., 2016). Furthermore, plant N content positively correlated with sediment porewater DIN concentration, which indicates that sediment DIN might be a major source for the plant's N acquisition in our experiment. Though previous studies showed that aquatic plants take up most of their P from the sediment *via* their roots (Carignan and Kalff, 1980), quite a few macrophytes, such as *V. americana*, *Heteranthera dubia*, *Myriophyllum spicatum*, and *M. alterniflorum* can take up a substantial amount of P from the water column *via* their shoots (Carignan and Kalff, 1980; Christiansen et al., 2016). In our study, plant P content increased with external nutrient loading, which confirms that the P from the water column was also an important source for the plant.

### Plant Elemental Composition and Stoichiometry

Even though the variance in plant C content was much lower than the variance in plant N and P contents, we did observe temperature and nutrient treatment effects on plant C content. In our study, plant C content decreased with rising temperature. The reason could be that rising temperature increased the growth of the plants and therefore depleted the C source, resulting in less inorganic C being available and less C in the plant tissue. Furthermore, with external nutrient loading, the growth of algae (both phytoplankton and periphyton) increased, and the algae may have competed for inorganic C with the submerged plants (Jones et al., 2002), thus leading to less inorganic C being available for the plants, resulting in a lower C content with external nutrient loading. Previous studies showed that increased C availability led to a decrease of N content of submerged plants (Madsen et al., 1998; Dülger et al., 2017). Our result is also in line with this observation as there was a negative covariation between plant C and N contents. This suggests that the plants can self-regulate their internal nutrient composition.

Plant N and P content (or C:N and C:P ratios) responded differently to rising temperature in nutrient-poor and nutrient-rich sediments. With rising temperature, there could be more dissolved inorganic N and P available for the plant in the environment, as rising temperature increases the mineralization rate of sediment organic matter (Gudasz et al., 2010; Sobek et al., 2017). In the nutrient-poor sediment, the growth of the plant might be limited by nutrients, as the DIP concentrations in the porewater were low (< 1 mg L^−1^) and DIN was almost 0 mg L^−1^. Therefore, the growth of the plant could mainly be limited by N at this condition. Hence, without external nutrient loading, the plant N content remained low and plant C:N ratio remained high across the whole temperature range (W0S0 treatment). At the meantime, plant P content might accumulate while the growth of the plant was limited, as P accumulation as a result of low growth rate has also been observed in a terrestrial shrub (Niu et al., 2019). The plant P content increased and C:P ratio decreased with rising temperature in nutrient-poor sediment, possibly because rising temperatures increased the availability of phosphate in the sediment, and plants accumulated more phosphorous at higher temperatures.

In contrast, in the nutrient-rich sediment, the growth of the plants did not seem to be limited by N or P. Without external nutrient loading (W0S1 treatment), the porewater DIN concentration increased with rising temperature, hence, the plant N content increased and C:N ratios decreased with rising temperature. However, the plant P content decreased and C:P ratio increased with nutrient enrichment in the sediment. The reason could be that the plants grew faster in the nutrient-rich sediment treatment, which dilutes the P content in the shoots and leads to a higher C:P ratio, as the plant invests the P in growth (Zhang et al., 2019).

A previous study using the same species found that both C:N and C:P ratios of *V. spiralis* increased with rising temperature (Zhang et al., 2019), as the plants grew longer and accumulated more biomass with rising temperature, which diluted the plant N and P content. Therefore, rising temperature could lead to a decreased, unaltered, or increased plant C:nutrient ratio. All in all, rising temperature could increase the nutrient availability in the sediment and the growth of the plant, thereby depleting the nutrients limiting plant growth and leading to an accumulation of non-limiting nutrients. It also matters where the nutrients were available, as nutrient enrichment of the sediment could enhance the growth of the plants, but nutrient enrichment of the water column can limit the growth of the plant by enhancing the growth of algae. External nutrient loading decreased plant C:nutrient ratios and this effect is independent of temperature (**Figure 7**). Therefore, we can conclude that temperature effects on aquatic plant C:nutrient ratios are not uniformly consistent, but highly dependent on the growth and the nutrient conditions (both N and P) in the environment, and they can either increase, remain unaltered or decrease.

### Plant Palatability

There were no detectable effects of temperature or nutrient treatments on plant palatability. Plant palatability was not correlated with any of the plant parameters that we measured. Feeding by herbivores can be determined by plant physical structure, plant nutrient level, and plant defence compounds (Cronin et al., 2002; Elger and Lemoine, 2005; Dorenbosch and Bakker, 2011). Even though a large amount of studies have shown that aquatic plant palatability might increase as plant N increases or C:N ratio decreases (Dorenbosch and Bakker, 2011; Bakker and Nolet, 2014; Bakker et al., 2016), not all studies find correlations between aquatic plant palatability and plant nutrient contents or stoichiometry (Cronin et al., 2002; Cronin and Lodge, 2003). Our result is in accordance with the latter. It might be that secondary metabolites which deterred the animals from feeding on the plants played a role in the feeding choice (Gross and Bakker, 2012; Agrawal and Weber, 2015; Grutters et al., 2017; Zhang et al., 2019). However, submerged plants are generally low in phenolic compounds (Smolders et al., 2000), the most common group of herbivore deterrent compounds in aquatic plants (Gross and Bakker, 2012). As the specific secondary compounds are largely unknown in freshwater aquatic plants (Gross and Bakker, 2012), we cannot further elaborate on their impacts on plant palatability here. Studies that find a correlation of plant palatability with plant physical and chemical traits are across a range of species (Elger and Willby, 2003; Elger and Barrat-Segretain, 2004; Dorenbosch and Bakker, 2011; Grutters et al., 2017). In contrast, studies that test one, or a few plant species generally did not observe such a relationship (Cronin et al., 2002; Cronin and Lodge, 2003). It seems that those traits of aquatic plants can better predict plant palatability at an inter-species level than an intra-species level (Zhang et al., 2019).

### Implications for Aquatic Ecosystems

Our study demonstrates that climate change and eutrophication could interactively alter the abundance and stoichiometry of aquatic plants. Even though we only chose one submerged rooted plant in our study, this species well represents other submerged-rooted macrophytes with the same strategy to take up nutrients from both the water column and the sediment (Carignan and Kalff, 1980; Rattray et al., 1991; Madsen and Cedergreen, 2002). Furthermore, aquatic plants representing other growth-forms, have a much simpler nutrient uptake strategy, either from the water column (floating plants), or from the sediment (emergent plants) (Guntenspergen et al., 1989). Therefore, our study can have broad implications for aquatic plants in general. A higher abundance of aquatic plants means a larger storage of C in the aquatic ecosystem (Fourqurean et al., 2012), and a shift in plant stoichiometry can be followed by a change in plant decomposition rate (Cebrian and Lartigue, 2004). Hence, changes in plant abundance and stoichiometry can have significant impacts on the nutrient cycling in aquatic plant-dominated ecosystems.

Although we did not observe that plant palatability changed under rising temperature and nutrient enrichment, nutrient enrichment has been shown to increase plant palatability in other aquatic plants, including *Potamogeton lucens* (Zhang et al., 2018b), which might result in enhanced top-down control of aquatic plants (Bakker and Nolet, 2014). Furthermore, plant-eating ectotherm herbivores can increase their consumption rate with warming as their metabolic rates increase (Zhang et al., 2018a), leading to enhanced top-down control of plants (O'connor, 2009; Schaum et al., 2018). Therefore, climate change and eutrophication might have strong impacts on aquatic plant-dominated ecosystems.

## Conclusions

We conclude that temperature rise and nutrient enrichment can have strong effects on aquatic plant growth and stoichiometry. Aquatic plant growth increased with rising temperature and nutrient enrichment in the sediment, whereas nutrient loading in the water column can inhibit the growth of the plant. The effects of temperature on plant stoichiometry highly depended on environmental nutrient conditions and plant growth. Despite alterations in plant stoichiometry with rising temperature, these changes did not alter plant consumption rates. Our results imply that warming and eutrophication can interactively alter plant abundances and stoichiometry, thereby influence the nutrient cycling in aquatic ecosystems.

## Data Availability Statement

The datasets generated for this study are available on request to the corresponding author. Data are available in the Dryad repository (doi: 10.5061/dryad.tqjq2bvv4).

## Author Contributions

PZ, MV, and EB formed the idea of the research and designed the experiment. PZ and AK conducted the experiment. PZ, CL, and JX did the data analysis. PZ, AK, CL, MV, ED, JX, and EB wrote and revised the paper.

## Funding

PZ acknowledges the China Scholarship Council (CSC) for funding his scholarship to study at NIOO-KNAW, and the China Postdoctoral Science Foundation (Grant No. 2019M652734) for supporting his postdoc research. The work of JX was supported by the National Key R&D Program of China (2018YFD0900904), the International Cooperation Project of the Chinese Academy of Sciences (Grant No. 152342KYSB20190025), the National Natural Science Foundations of China (Grant No. 31872687), and the Water Pollution Control and Management Project of China (Grant No.2018ZX07208005). The work of MV is funded by the Gieskes-Strijbis Foundation and the International IGB Fellowship Program “Freshwater Science” of the Leibniz‐Institute of Freshwater Ecology and Inland Fisheries.

## Conflict of Interest

The authors declare that the research was conducted in the absence of any commercial or financial relationships that could be construed as a potential conflict of interest.
