# Supplementary material for: Interactive Effects of Rising Temperature and Nutrient Enrichment on Aquatic Plant Growth, Stoichiometry, and Palatability
Source: Front Plant Sci. 2020 Feb 12;11:58. doi: 10.3389/fpls.2020.00058 (PMC7028819; doi:10.3389/fpls.2020.00058)
Supplement: Supplementary file 1 [file Table_1.docx]

**
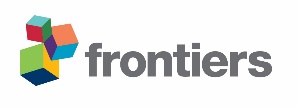
**

***Supplementary Material***

**Supplementary Material includes the information below:**

**Figure S1** Experimental design and layout.

**Figure S2** Water quality measurements in the water column over time during the experiment indicated per nutrient treatment.

**Figure S3** Temperature effects on nutrient concentrations in the sediment porewater and algae growth at the end of the experiment, indicated per nutrient treatment.

**Table S1** Effects of temperature, sediment type, external nutrient loading and their interactions on algae growth and nutrient concentrations in the sediment porewater at the end of the experiment.

**R code for the structure equation model**

**Figure S1** **Experimental design and layout.** (a) Plant in each vase; (b) Layout of the four different nutrient treatments in one aquarium, the distribution was random within each aquarium. S1 indicates nutrient-rich sediment, S0 indicates nutrient-poor sediment, W1 indicates external nutrient loading to the water, and W0 indicates without external nutrient loading. (c) Side view of the set-up at the beginning of the experiment.


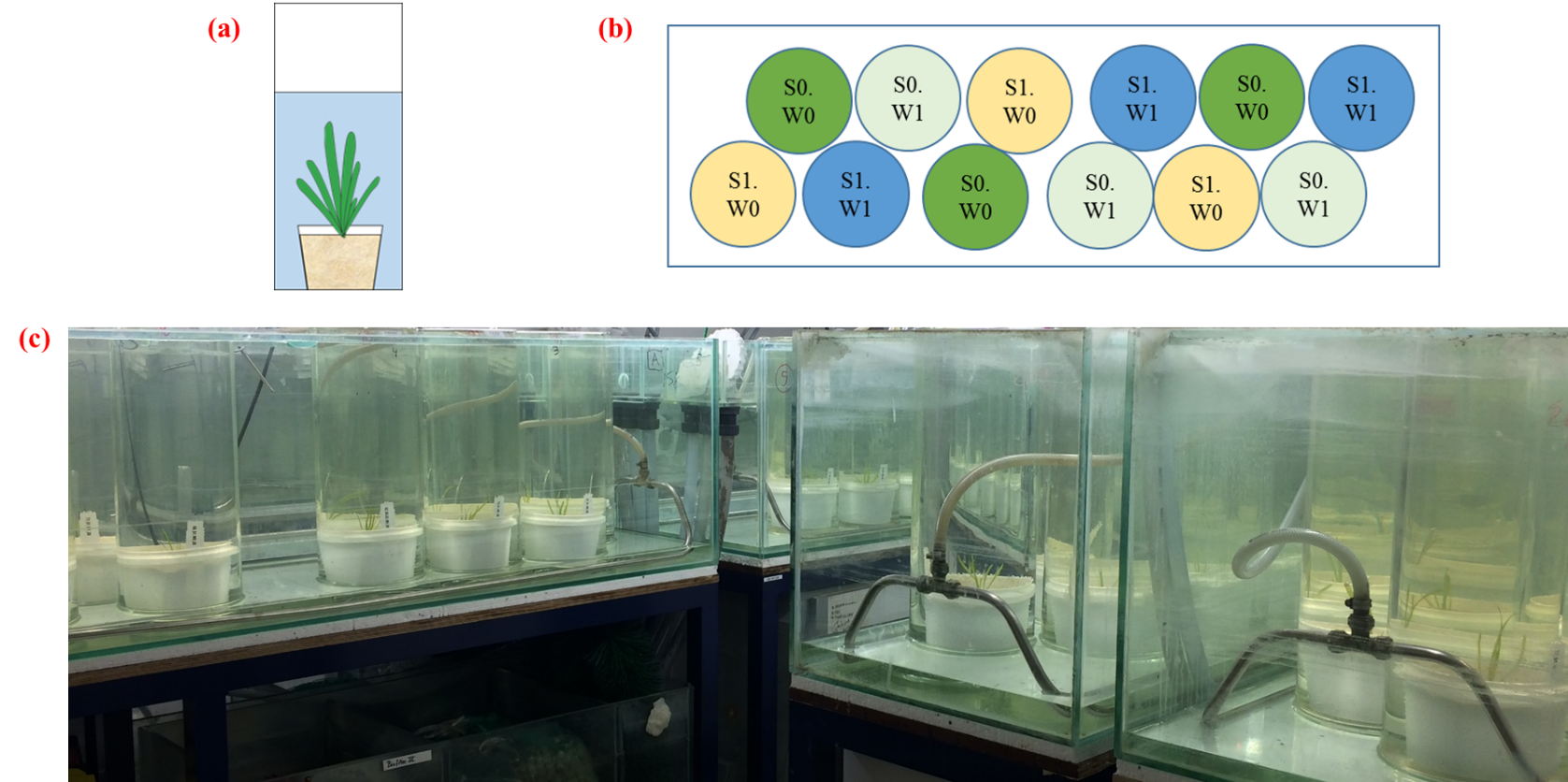


**Figure S2** **Water quality measurements in the water column over time during the experiment indicated per nutrient treatment.** Parameters include conductivity, pH, Chlorophyll a, alkalinity, DIN (the sum of N from NH_4_^+^, NO_2_^-^ and NO_3_^-^)and P-PO_4_^3-^ (the P from PO_4_^3-^). Nutrient treatments are as indicated in Fig. S1.

Conductivity and pH were measured with a multi–meter (Multi 350i/SET, Germany). Alkalinity was measured by an auto-titration machine (TIM840 titration manager, Germany). Chlorophyll a (Chl a) concentration in the water column was determined by chlorophyll fluorescence on a phytoplankton analyser (PHYTO-PAM, WALZ, Germany). Ammonium (NH_4_^+^), nitrite (NO_2_^-^), nitrate (NO_3_^-^) and orthophosphate (PO_4_^3-^) were analyzed by an AutoAnalyzer (QuAAtro, Seal Analytical, Fareham, UK) after filtering water samples over prewashed GF/F filter (Whatman, Maidstone, UK).


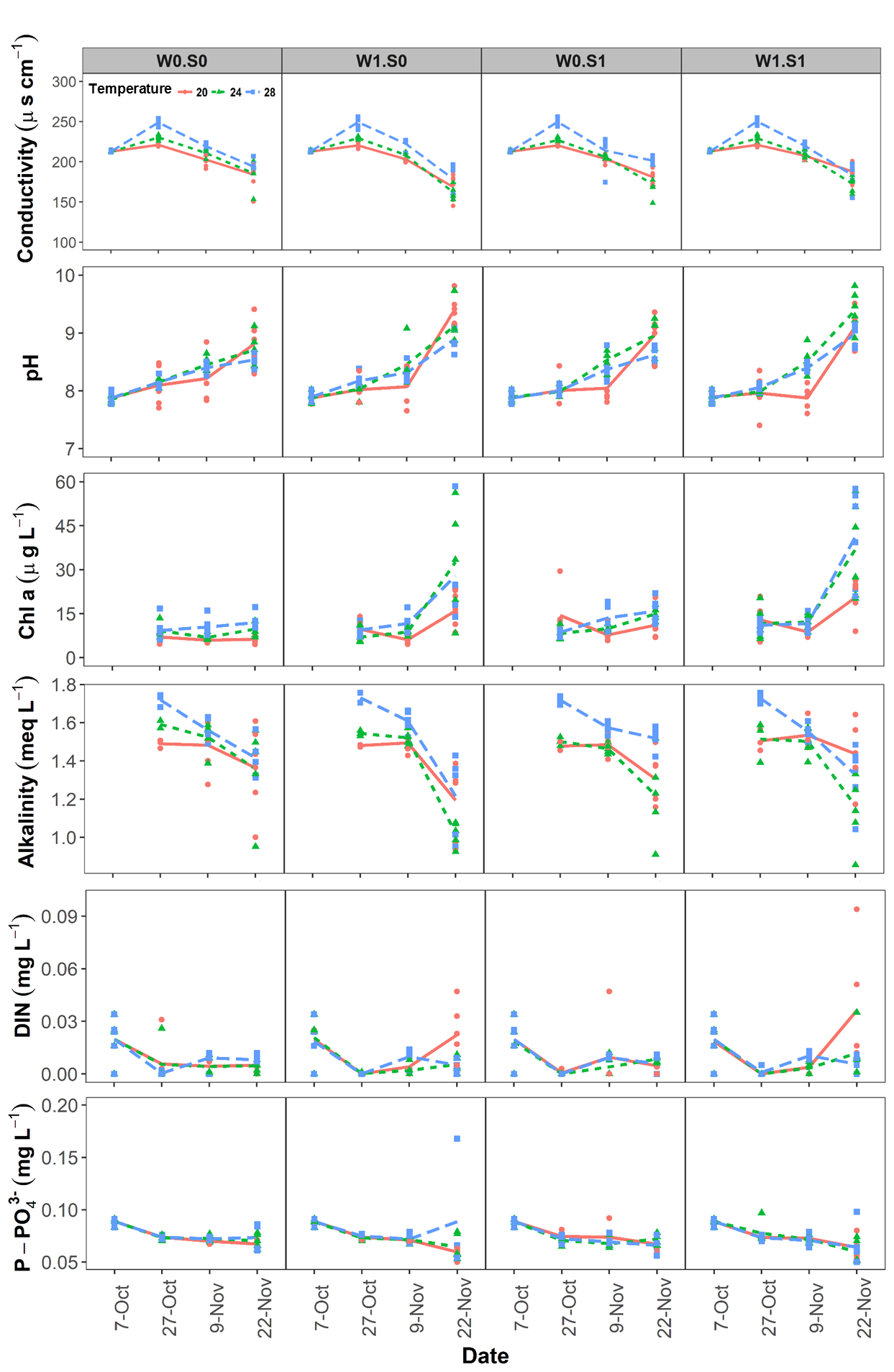


**Figure S3** **Temperature effects on nutrient concentrations in the sediment porewater and algae growth at the end of the experiment, indicated per nutrient treatment.** DIN indicates total dissolved inorganic nitrogen (sum of N-NH_4_^+^, N-NO_2_^-^ and N-NO_3_^-^). Nutrient treatments are as indicated in Fig. S1. A solid line indicates *p* < 0.05. Statistic results are indicated in Table S1.


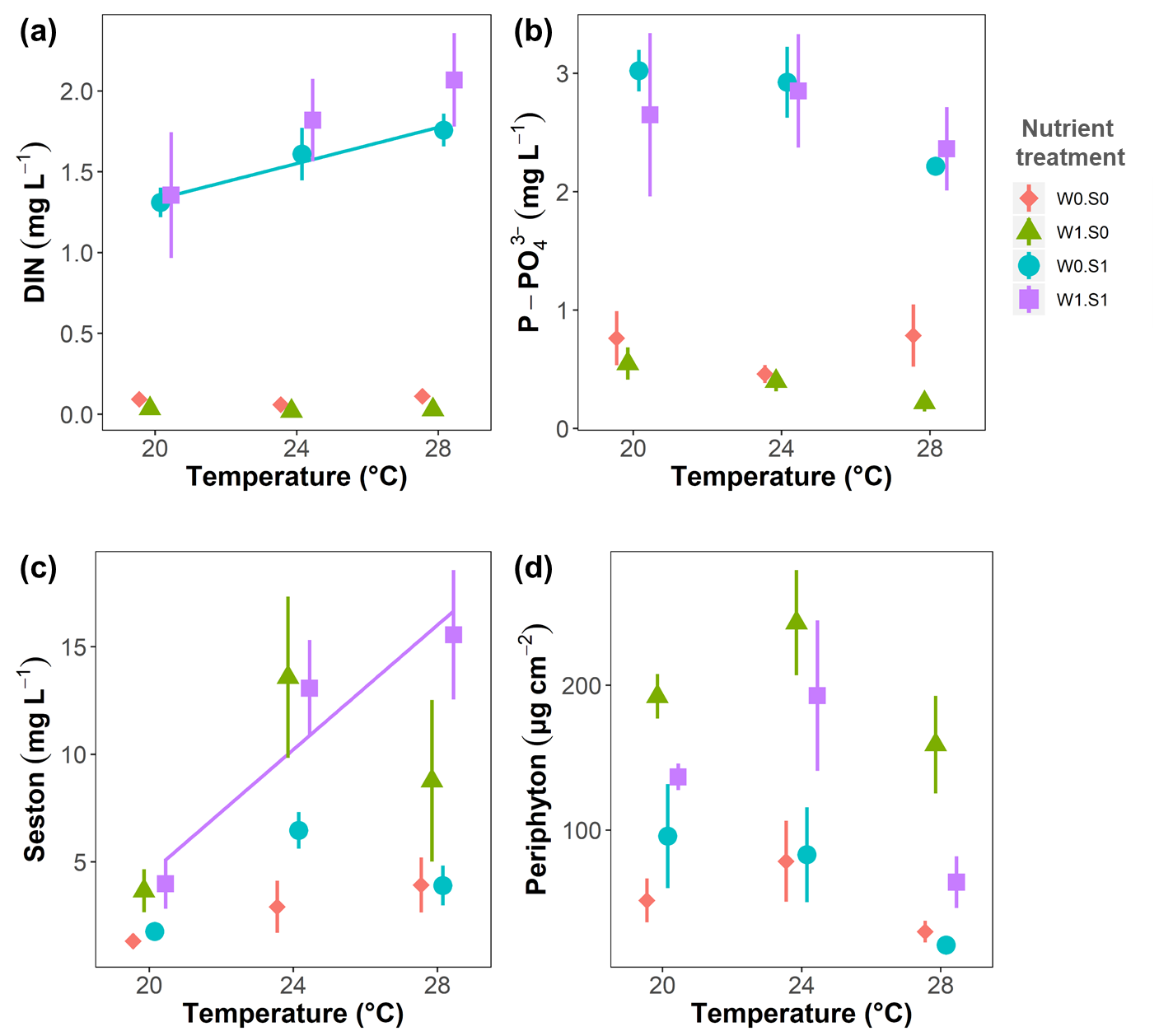


**Table S1 Effects of temperature, nutrient treatment and their interactions on algae growth, and nutrient concentrations in the sediment porewater at the end of the experiment.** Effects were tested by Linear-Mixed Effect models. Data transformation to meet model requirements is indicated.

| **Category** | **Parameters** | **Factors** | **df** | ***F*** | ***p*-value** | **Means comparsion** | **Slopes comparsion** |
| --- | --- | --- | --- | --- | --- | --- | --- |
| **Algae growth** | **Seston** | Temp | 1, 4 | 3.98 | 0.1167 | a, b, a, b | A, A, A, A |
|  |  | Nutrient | 3, 55 | 13.00 | **<0.0001** |  |  |
|  |  | Temp × Nutrient | 3, 55 | 2.79 | **0.0489** |  |  |
|  | **sqrt(Periphyton)** | Temp | 1, 4 | 2.46 | 0.1916 | a, c, a, b | A, A, A, A |
|  |  | Nutrient | 3, 55 | 23.10 | **<0.0001** |  |  |
|  |  | Temp × Nutrient | 3, 55 | 0.86 | 0.4668 |  |  |
| **Nutrients in the pore water** | **log(DIN + 0.01)** | Temp | 1, 4 | 4.99 | 0.0892 | a, b, c, c | A, A, A, A |
|  |  | Nutrient | 3, 55 | 166.62 | **<0.0001** |  |  |
|  |  | Temp × Nutrient | 3, 55 | 0.16 | 0.9214 |  |  |
|  | **P-PO_4_^3-^** | Temp | 1, 4 | 0.74 | 0.4368 | a, a, b, b | A, A, A, A |
|  |  | Nutrient | 3, 55 | 59.73 | **<0.0001** |  |  |
|  |  | Temp × Nutrient | 3, 55 | 0.81 | 0.4928 |  |  |

Means and slopes comparison among the four nutrient treatments were performed after each linear mixed-effect model test. Different letters indicate difference among the four nutrient treatments in an order of W0.S0, W1.S0, W0.S1 and W1.S1, same with the order present in Fig.2,3&4. “Temp” represents temperature treatment. “Nutrient” indicates the four nutrient treatments. DIN means total dissolved inorganic nitrogen (including N from NH_4_^+^, NO_2_^-^ and NO_3_^-^). “log” and “sqrt” indicate the data are natural log and square root transformed respectively. Bold numbers indicate *p <* 0.05.

**R code for the structure equation model**

#import the data#

Alldata_sem <- read.delim("Alldata_sem.txt")

Alldata_sem <- subset(Alldata_sem, Dead_plant == 0) #select data, remove the dead plants

library(lavaan)

Alldata_sem$Growthrate <- Alldata_sem$Growthrate * 100 # increase the magnitude of variances

Alldata_sem$Periphyton <- Alldata_sem$Periphyton / 100 # decrease the magnitude of variances

#construct the model

Vmodel <- '

# Lavaan regression

Growthrate ~ Sediment + Temp + Periphyton

Periphyton ~ Water + Temp

Ccon ~ Sediment + Water + Temp + Growthrate

Ncon ~ Sediment + Water

Pcon ~ Water + Temp + Growthrate

'

fit <- sem(Vmodel, data=Alldata_sem,estimator = "MLR", missing = "ml")

summary(fit, fit.measures=TRUE, standardized=TRUE,modindices = FALSE, rsquare = T)
